# Emotional distress is associated with neuroendocrine-immune remodeling and less favorable neoadjuvant immunotherapy outcomes in oral squamous cell carcinoma

**DOI:** 10.3389/fimmu.2026.1861003

**Published:** 2026-07-01

**Authors:** Ze-Chen Zhao, Xi Huang, Xue-Peng Xiong, Wei Zhang, Zi-Li Yu, Jun Jia, Bing Liu, Jian-Gang Ren, Gang Chen

**Affiliations:** 1 State Key Laboratory of Oral & Maxillofacial Reconstruction and Regeneration, Key Laboratory of Oral Biomedicine Ministry of Education, Hubei Key Laboratory of Stomatology, School & Hospital of Stomatology, Wuhan University, Wuhan, China; 2 Department of Oral and Maxillofacial Surgery, School and Hospital of Stomatology, Wuhan University, Wuhan, China; 3 TaiKang Center for Life and Medical Sciences, Wuhan University, Wuhan, China; 4 Frontier Science Center for Immunology and Metabolism, Wuhan University, Wuhan, China

**Keywords:** emotional distress, macrophage-Treg interaction, neoadjuvant immunotherapy, oral squamous cell carcinoma, tumor microenvironment

## Abstract

**Background:**

Immunotherapy research, particularly in the neoadjuvant setting, has largely focused on tumor-intrinsic determinants, with limited attention to host-related factors. Emotional distress (ED), a prevalent condition in cancer patients, remains poorly understood in the context of neoadjuvant immunotherapy (NAIT).

**Methods:**

We analyzed a cohort of 68 patients with locally advanced OSCC treated with NAIT. ED was assessed using the EORTC QLQ-C30 to stratify patients, and clinical outcomes were evaluated to determine therapeutic efficacy. Serum neuroendocrine markers were measured to assess neuroendocrine activation. Integrated single-cell, spatial, and multiplexed imaging approaches were employed to characterize immune remodeling and macrophage-Treg interactions. A chronic restraint stress mouse model was established to investigate stress-associated tumor immune remodeling and therapeutic response during anti-PD-1 therapy.

**Results:**

Elevated ED was associated with a lower disease control rate and inferior event-free survival. High-distress patients exhibited increased serum cortisol levels, which were associated with less favorable treatment outcomes. Multi-omics analyses suggested an immunosuppressive tumor microenvironment in distressed patients, characterized by regulatory T cell enrichment and increased macrophage–Treg spatial association within organized niches. *In vivo*, RU486 treatment was associated with reduced tumor burden, improved survival, and partial modulation of immunosuppressive remodeling.

**Conclusions:**

Emotional distress may represent a clinically relevant and potentially modifiable host-related factor associated with NAIT outcomes in OSCC. Stress-associated glucocorticoid signaling may contribute to immunosuppressive tumor remodeling and less favorable therapeutic response. These findings provide a rationale for integrating psychosocial assessment and neuroendocrine-targeted interventions into perioperative immunotherapy strategies.

## 1 Introduction

Oral squamous cell carcinoma (OSCC) is a biologically aggressive malignancy marked by substantial intratumoral heterogeneity and complex immune dynamics (1). Despite advances in multimodal therapeutic regimens integrating surgery, radiotherapy, and chemotherapy, survival improvements have been disappointing, particularly among patients with locally advanced disease (2, 3). Persistent recurrence and metastatic progression emphasize the critical need to improve response prediction and develop transformative therapeutic strategies (4).

In recent years, neoadjuvant immunotherapy (NAIT), particularly PD-1/PD-L1 blockade, has emerged as a promising therapeutic strategy for patients with advanced OSCC (5, 6). However, durable clinical benefit remains restricted to a subset of patients, as a considerable proportion fail to achieve sustained responses (7, 8). This substantial inter-individual variability underscores the limitations of current predictive biomarkers in reliably stratifying candidates for immunotherapy. Although PD-L1 expression is widely used for therapeutic guidance, its prognostic and predictive relevance in OSCC remains controversial (9). These reports suggest that tumor-intrinsic features such as PD-L1 expression and tumor mutational burden (TMB) are insufficient to fully account for the observed variability in clinical outcomes. Increasing evidence indicates that host-related factors, including neuroendocrine regulation, metabolic state, and psychosocial conditions, may shape antitumor immune responses and thereby influence treatment outcomes (10, 11).

Emotional distress (ED), a sustained negative psychological state induced by stressors, is common among patients with cancer (12, 13). Clinical studies have shown that higher stress levels are associated with shorter progression-free survival in patients with metastatic melanoma and non-small cell lung cancer treated with anti-PD-1 therapy (14–16), suggesting that psychological stress may be linked to altered antitumor immunity and immunotherapeutic outcome. Notably, OSCC arises within the oral mucosa, a tissue that is richly innervated and densely populated with immune cells, providing a unique neuro-immune interface (17–19). As immune checkpoint inhibitors become increasingly incorporated into OSCC treatment, understanding whether emotional distress modulates therapeutic response is of growing clinical relevance. However, the mechanistic impact and clinical significance of emotional distress in the context of NAIT for OSCC remain largely undefined.

In this study, leveraging our phase II clinical trial (NCT04649476), we systematically evaluated the association between baseline emotional distress and NAIT outcome in patients with locally advanced OSCC. By integrating single-cell transcriptomic profiling with clinical data, we evaluated the clinical association between baseline emotional distress and NAIT outcomes and explored related alterations within the tumor immune microenvironment. Our findings suggest a potential link between psychological stress, tumor immune remodeling, and less favorable immunotherapeutic outcomes in OSCC, offering a rationale for integrating psychosocial modulation into combinatorial treatment strategies.

## 2 Materials and methods

### 2.1 Study design and participants

This study is a *post hoc* analysis of prospectively collected data from a previously registered clinical trial evaluating neoadjuvant anti-PD-1 immunotherapy with or without chemotherapy in patients with OSCC (NCT04649476). Patients were enrolled between 2021 and 2022. The trial protocol, including psychological assessment and biospecimen collection for translational research, was approved by the Institutional Ethics Committee of the School and Hospital of Stomatology, Wuhan University ([2020] Ethics No.2). Written informed consent was obtained from all participants.

Eligible patients had resectable stage III-IVA OSCC according to the AJCC 8th edition staging system and had received no prior systemic therapy. All patients received intravenous camrelizumab 200 mg on days 1, 15, and 29 before surgery. Patients in the combination-therapy group additionally received TPF chemotherapy, consisting of docetaxel 75 mg/m², cisplatin 75 mg/m², and 5-fluorouracil 750 mg/m² intravenously on days 1–5 and 22-26. Postoperative management followed NCCN Guidelines for Head and Neck Cancers (v2.2018). Radiological response was evaluated in 66 response-evaluable patients, including 30 patients with ED and 36 patients without ED. Disease control rate (DCR) was defined as the proportion of response-evaluable patients achieving complete response, partial response, or stable disease. Event-free survival (EFS) was measured from the first dose of neoadjuvant therapy to the first occurrence of disease progression, recurrence, death from any cause, or last follow-up for censored patients. Disease-free survival (DFS) was defined as the time from definitive surgery to the first documented local recurrence, regional recurrence, distant metastasis, or death from any cause. Patients without DFS events were censored at the date of last follow-up. The median follow-up time was 30.24 months.

### 2.2 Assessment of emotional distress

Emotional functioning was assessed at baseline before NAIT using the emotional functioning subscale of the European Organisation for Research and Treatment of Cancer Quality of Life Questionnaire Core 30 (EORTC QLQ-C30). This domain includes four items assessing whether patients felt tense, worried, irritable, or depressed. After linear transformation, scores range from 0 to 100, with higher scores indicating better emotional functioning. Based on previously established thresholds for clinical importance for the EORTC QLQ-C30 emotional functioning domain, patients were categorized into an emotionally preserved group (>71) and an emotionally impaired group (≤71) (16). In the present study, the emotionally impaired group was referred to as the emotional distress (ED) group, whereas the emotionally preserved group was referred to as the No-ED group. This classification reflects patient-reported emotional functioning impairment rather than a formal psychiatric diagnosis.

### 2.3 Immunohistochemistry

Immunohistochemical (IHC) staining was performed on formalin-fixed, paraffin-embedded tissue sections according to standard protocols. The following primary antibodies were used for human tissue staining: CD3 (CST, #85061S, 1:200), CD4 (Abcam, ab133616, 1:400), CD8 (CST, #85336S, 1:400), FOXP3 (CST, #98377S, 1:100), CD20 (Abcam, ab64088, 1:200), CD68 (CST, #76437S, 1:400), CD14 (Abcam, ab182032, 1:400), CD11c (Abcam, ab56232, 1:200). After staining, whole-slide images and quantitative analysis were acquired using Leica digital pathology scanning system.

### 2.4 Raw data processing and scRNA-seq analysis

Raw single-cell RNA sequencing (scRNA-seq) reads were processed using the CeleScope pipeline (v1.9.0) to generate gene-cell expression matrices. Briefly, adaptor sequences and poly-A tails were trimmed using Cutadapt (v1.17), and reads failing quality thresholds were discarded. Cell barcodes and unique molecular identifiers (UMIs) were extracted prior to alignment. Cleaned reads were aligned to the human reference genome (GRCh38) using STAR (v2.6.1a), and gene-level count matrices were generated with featureCounts (v2.0.1).

Downstream analyses were performed using Scanpy (v1.9.8). Quality control filtering excluded cells expressing fewer than 200 genes or more than 5,500 genes, cells with mitochondrial gene content exceeding 20%, and genes detected in fewer than three cells. After filtering, 125,593 high-quality cells were retained for subsequent analyses. Count data were normalized to total counts per cell and log-transformed. Highly variable genes were identified, and principal component analysis (PCA) was conducted on the scaled expression matrix. To mitigate batch effects across samples, datasets were integrated using the batch balanced k-nearest neighbors (BBKNN) algorithm, which constructs a batch-corrected neighborhood graph by identifying cross-batch nearest neighbors. The top 50 principal components were used for neighborhood graph construction, clustering, and dimensionality reduction. Uniform Manifold Approximation and Projection (UMAP) was applied for visualization. For single-cell analyses, cell proportion comparisons were performed using patients as the biological unit whenever applicable.

### 2.5 Cell-cell communication analysis

Cell-cell communication networks were inferred using the CellChat R package (v1.6.1). Normalized gene expression matrices and cell-type annotations derived from single-cell RNA sequencing analysis were used as input. Ligand-receptor interactions were predicted based on the curated CellChat database of known signaling interactions. Communication probability between cell types was calculated using the default probabilistic model, which integrates average gene expression levels and the proportion of expressing cells. Signaling pathways were aggregated at the pathway level to quantify overall communication strength. Visualization of communication networks was conducted using built-in functions, including circle plots, heatmaps, and bubble plots.

### 2.6 Animals

Female C57BL/6 mice (6–8 weeks old) were maintained in a specific pathogen-free (SPF) facility under controlled conditions (22-25 °C, 12 h light/dark cycle) with free access to standard chow and water. All mice were acclimated to the environment for 1 week prior to experimentation. To establish subcutaneous OSCC models, 5 × 10^5^ MOC2 cells were inoculated subcutaneously into the right dorsal flank of mice (designated as Day 0). All mice were then subjected to chronic restraint stress (CRS) for 21 consecutive days (20). During stress procedures, mice were restrained in well-ventilated plastic holders for 3 h per day without food or water.

For therapeutic interventions, tumor-bearing mice were randomly allocated into four groups (n=5): isotype control (IgG), GR inhibitor (RU486) + IgG, anti-PD-1 monotherapy, and RU486 + anti-PD-1 combination therapy. Treatments included: 200 μg/mouse IgG isotype control antibody (Bio X Cell, #BE0083), 200 μg/mouse anti-PD-1 antibody (Bio X Cell, #BE0273), and RU486 (MCE, HY-13683) at 10 mg/kg daily by intraperitoneal administration. All antibodies and RU486 were administered via intraperitoneal injection according to the indicated schedule. Tumor size was measured every other day using a digital caliper, and tumor volume was calculated using the formula (length × width²)/2. Tumor measurements and outcome assessments were performed by investigators blinded to group allocation. Mice were euthanized when the maximum tumor diameter reached 1.5 cm or at the predetermined endpoint for tumor volume and weight analysis. Animals were first anesthetized with isoflurane (2-3% in oxygen) and subsequently euthanized by CO_2_ inhalation at a flow rate of 20-30% of chamber volume per minute. For survival analysis, mice were monitored until humane endpoints were reached. All animal procedures were approved by the Institutional Animal Care and Use Committee of the School of Stomatology (Approval No.S07923050A), Wuhan University and performed following ARRIVE guidelines.

### 2.7 Multiplex immunohistochemistry

Multiplex immunohistochemistry was performed using the Opal 6-Plex Detection Kit (PerkinElmer, NEL861001KT) following the manufacturer’s protocol. Formalin-fixed, paraffin-embedded (FFPE) tissue sections (4 μm) were deparaffinized in xylene and rehydrated through graded ethanol solutions. After rehydration, slides were fixed in 10% neutral buffered formalin and subjected to heat-induced antigen retrieval in EDTA buffer. The following primary antibodies were used for human tissue staining: pan-cytokeratin (CST, #4545, 1:400, Opal 650), CD68 (CST, #76437S, 1:400, Opal 520) and FOXP3 (CST, #98377S, 1:100, Opal 620). For mouse tissue staining, the following primary antibodies were used: pan-cytokeratin (CST, #4545, 1:400, Opal 650), F4/80 (CST, #70076, 1:400, Opal 520), Arg1 (CST, #93668, 1:200, Opal 570), FOXP3 (Proteintech, 22228-1-AP, 1:400, Opal 620), Ki67 (CST, #12202, 1:200, Opal 690). Antibody binding was detected using polymer HRP conjugates followed by tyramide signal amplification with Opal fluorophores. Nuclei were counterstained with DAPI. Multispectral images were acquired using the Vectra quantitative pathology imaging system (Akoya Biosciences, USA) and processed using the corresponding image analysis software for cell phenotyping and spatial quantification.

### 2.8 Measurement of plasma ACTH and cortisol

Blood samples for adrenocorticotropic hormone (ACTH) and cortisol measurement were collected before the NAIT initiation. To minimize circadian and dietary variability, all samples were obtained uniformly at 8:00 AM under morning fasting conditions. Peripheral blood was collected into EDTA anticoagulant tubes, immediately processed for plasma separation, aliquoted, and stored under standardized conditions until analysis. Plasma ACTH and cortisol levels were measured using a standardized clinical laboratory assay platform according to the manufacturer’s instructions. Medication history was reviewed before and during treatment. Patients with active systemic infection, recent systemic corticosteroid exposure, psychotropic medication use, or substance abuse were excluded according to the trial protocol.

### 2.9 Statistical analysis

Statistical analyses were performed using R software (v4.3.2) and GraphPad Prism (v9.0). Continuous variables were assessed for normality using the Shapiro–Wilk test. Normally distributed variables were compared using two-tailed Student’s t-test, whereas non-normally distributed variables were compared using the Mann–Whitney U test. Categorical variables were compared using the χ² test. For clinical outcome analyses, disease control was evaluated among radiologically evaluable patients, whereas baseline characteristics and survival analyses were evaluated in the full 68-patient cohort. Multivariable logistic regression was used to examine whether baseline clinical variables were associated with ED status, with Firth penalized logistic regression performed as a sensitivity analysis. Cox proportional hazards regression was used for time-to-event outcomes. Kaplan–Meier curves were compared using the log-rank test. Data are presented as mean ± SEM or median with interquartile range. Each dot represents an independent patient, mouse or biological replicate. P < 0.05 was considered statistically significant.

## 3 Results

### 3.1 Emotional distress is associated with poorer clinical outcomes in patients receiving NAIT

**Figure 1:**
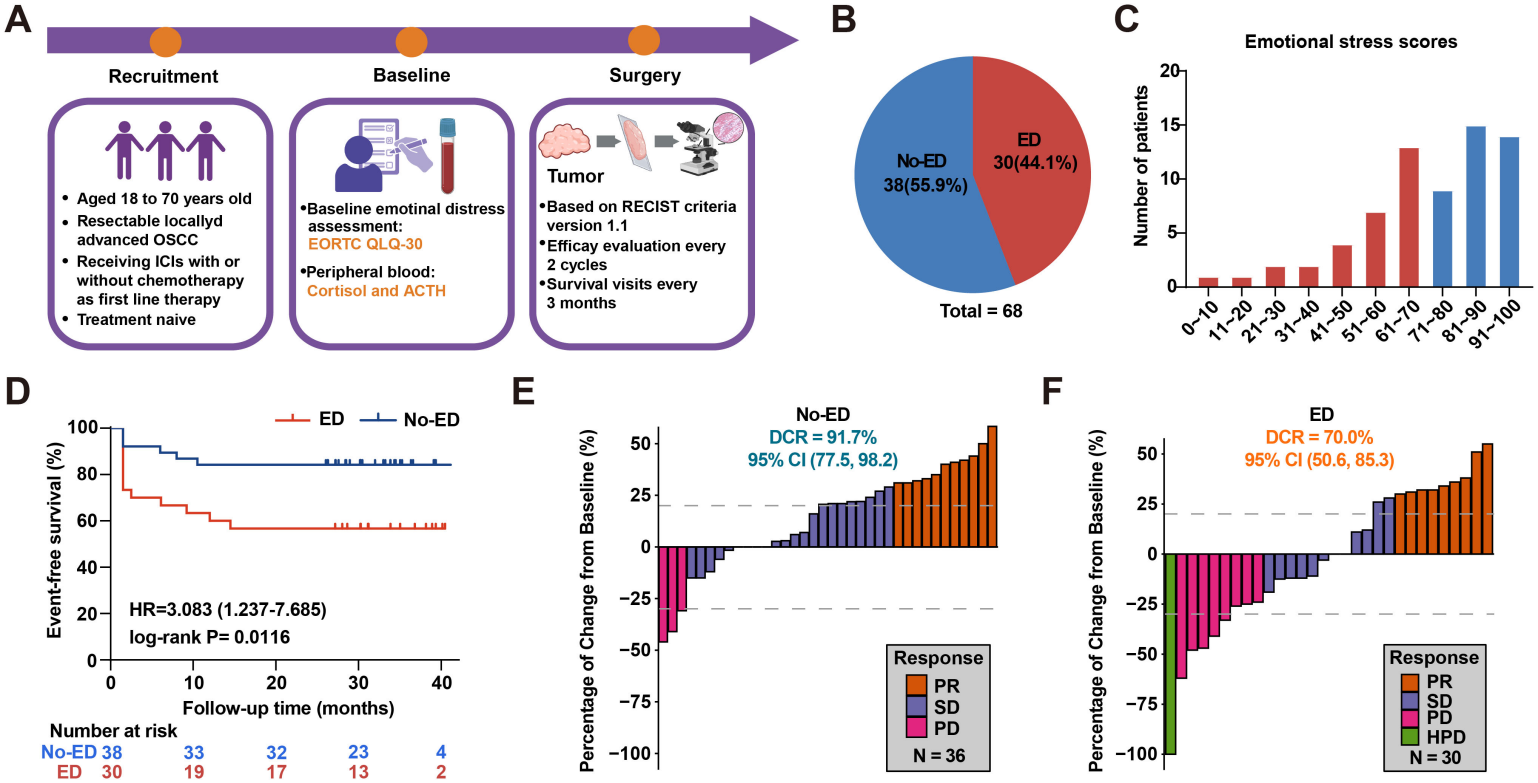
Emotional distress is associated with poorer clinical outcomes in patients receiving NAIT. **(A)** Workflow of the clinical trial, emotional distress assessment, and clinical response evaluation. **(B)** Distribution of emotional distress status among 68 patients with OSCC. **(C)** Number distribution of patients across different levels of emotional distress. **(D)** Kaplan–Meier analysis of event-free survival in patients with and without baseline emotional distress (n=68). **(E)** Waterfall plot showing the maximal percentage change of target lesions after NAIT in radiologically evaluable patients without emotional distress (No-ED, n=36). **(F)** Waterfall plot showing the maximal percentage change of target lesions after NAIT in radiologically evaluable patients with emotional distress (ED, n=30). Statistical significance for EFS was assessed using the log-rank test.

We analyzed a cohort of OSCC patients treated with neoadjuvant immunotherapy within a clinical trial (NCT04649476) to examine the relationship between emotional distress and treatment outcomes (**Figure 1A**). Among 68 patients, 30 (44.1%) were classified as emotionally distressed, with 20% exhibiting severe impairment (**Figures 1B, C**). Baseline clinical characteristics were broadly comparable between the ED and No-ED groups (**Supplementary Table 1**). Multivariable logistic regression and Firth penalized logistic regression showed that treatment regimen, betel nut use, and other examined clinical variables were not significantly associated with ED status in the multivariable models, suggesting that the ED grouping was unlikely to be primarily explained by these baseline factors (**Supplementary Tables 2**, **3**). Additionally, we performed a multivariable Cox regression for event-free survival (EFS), incorporating treatment regimen and other clinically relevant covariates. Even after accounting for these variables, the association between ED and EFS remained evident (**Supplementary Table 4**). Kaplan–Meier analysis showed that patients with emotional distress had significantly worse event-free survival than those without emotional distress (HR = 3.083, 95%CI 1.24-7.69; P = 0.0116) (**Figure 1D**). Among 66 radiologically evaluable patients, including 36 No-ED patients and 30 ED patients, No-ED patients exhibited a higher disease control rate than those with distress (DCR: 33/36, 91.7% vs. 21/30, 70.0%) (**Figures 1E, F**). Moreover, progressive disease (PD) and hyperprogressive disease (HPD) were also more commonly observed in the ED group (**Figures 1E, F**). In contrast, patients without emotional distress were more likely to achieve deeper tumor regression. Of note, ROC analysis showed that emotional distress had modest discriminatory ability for complete or partial response to NAIT (AUC = 0.62; 95%CI 0.418-0.759; P = 0.09). Although this result did not reach statistical significance, the observed trend warrants validation in larger cohorts (**Supplementary Figure 1**). Together, these findings suggest a potential association between baseline emotional distress and less favorable NAIT outcomes in patients with OSCC.

### 3.2 Regulatory T cell enrichment in the tumor microenvironment of emotionally distressed patients

**Figure 2:**
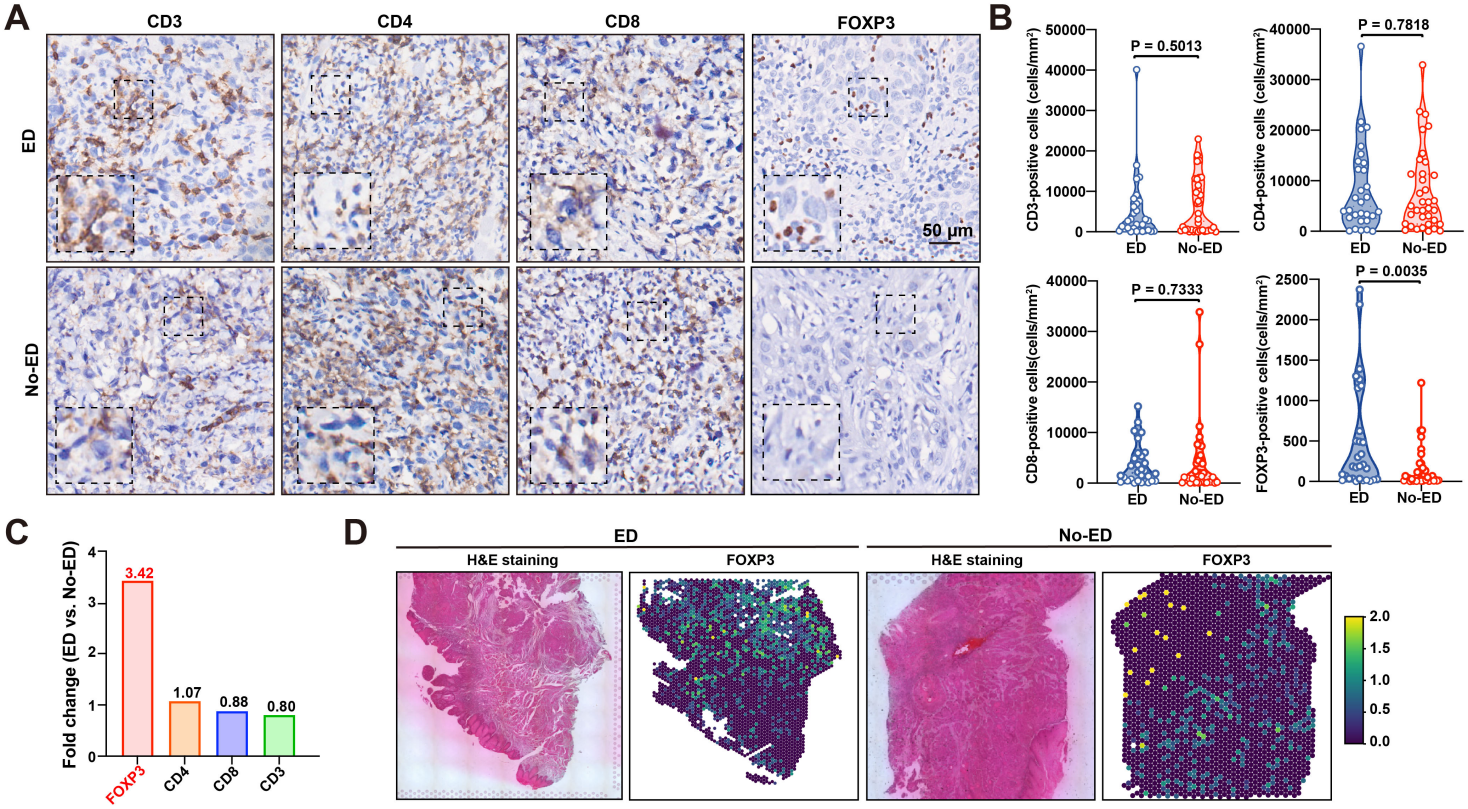
Regulatory T cell enrichment in the tumor microenvironment of emotionally distressed patients. **(A)** Representative immunohistochemical staining of CD3, CD4, CD8, and FOXP3 in tumor regions. **(B)** Quantitative comparison of immune cell infiltration between ED and No-ED groups. **(C)** Fold change analysis of immune cell populations between ED and No-ED groups. **(D)** Spatial transcriptomic analysis showing FOXP3 expression in tumor regions across patients with different emotional distress levels. Statistical significance was assessed using two-tailed Student’s t-test or Mann–Whitney U test according to data distribution.

To determine whether emotional distress alters the tumor immune microenvironment, we analyzed immune cell infiltration in tumors from ED and No-ED patients using immunohistochemistry. Representative staining of CD3, CD4, CD8, and FOXP3 revealed distinct immune infiltration patterns across emotional distress groups (**Figure 2A**). Interestingly, although CD3+, CD4+, and CD8+ T lymphocytes were abundantly present in tumors, their infiltration levels were comparable between the two groups. We observed that FOXP3+ cells were significantly increased in tumors from patients with emotional distress. Quantitative analysis further confirmed significant differences in immune cell infiltration between ED and No-ED groups (**Figure 2B**). In particular, FOXP3+ regulatory T cells (Tregs) were markedly enriched in the ED group, with a 3.42-fold increase compared with the No-ED group (**Figure 2C**). Consistent with these findings, spatial transcriptomic analysis revealed enhanced FOXP3 expression in tumor regions from patients with higher levels of emotional distress (**Figure 2D**). We also evaluated the infiltration of additional immune cell subsets within the tumor microenvironment, including CD14+ monocytes, CD20+ B cells, CD68+ macrophages, and CD11c+ dendritic cells (**Supplementary Figure 2**). However, no significant differences were observed between ED and No-ED groups for these immune populations. Collectively, these observations suggest that emotional distress is associated with remodeling of the tumor immune landscape toward an immunosuppressive microenvironment, primarily characterized by regulatory T cell enrichment.

### 3.3 Regulatory T cells from emotionally distressed patients exhibit distinct immunoregulatory features in OSCC

**Figure 3:**
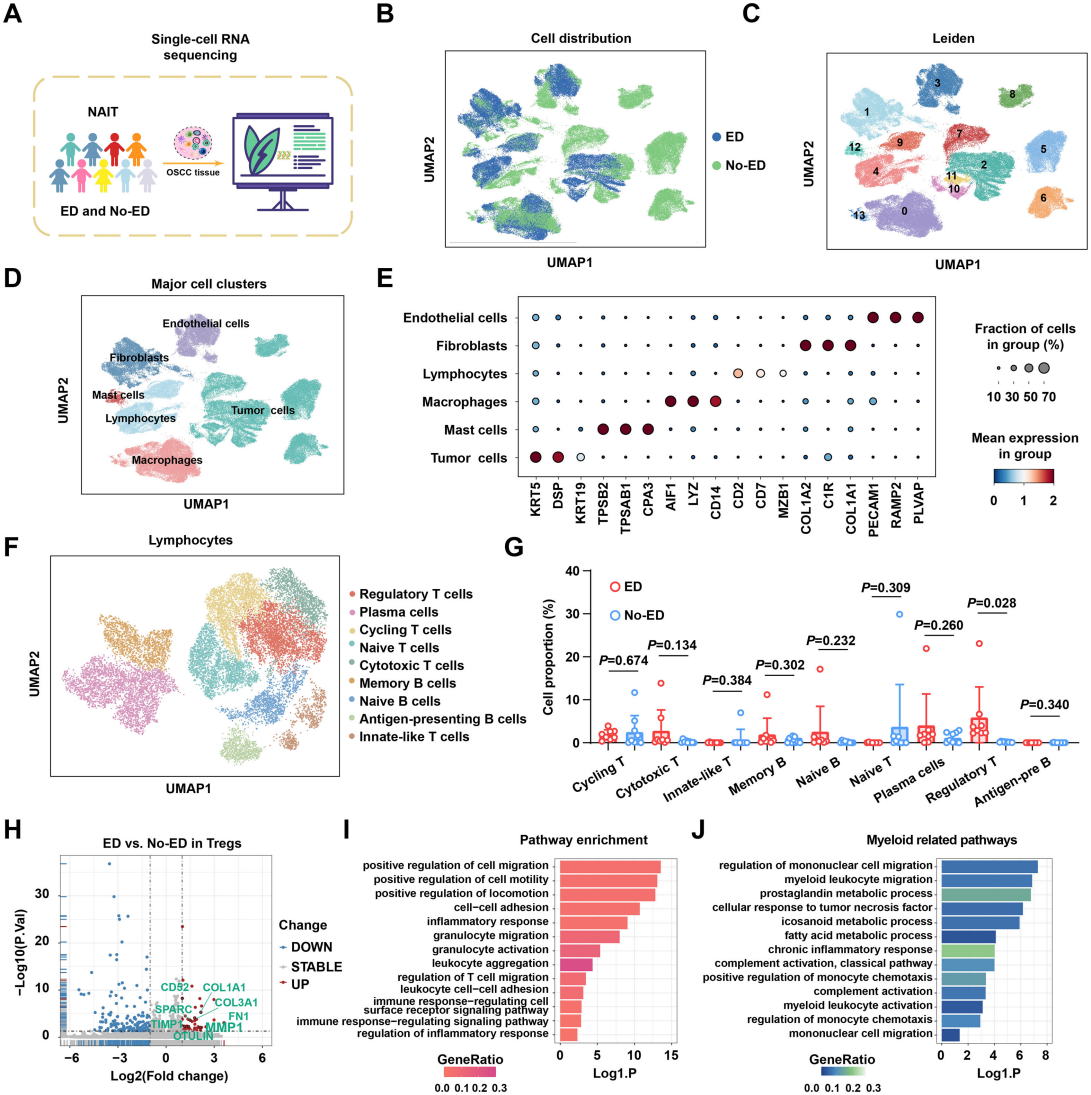
Regulatory T cells from emotionally distressed patients exhibit distinct immunoregulatory features in OSCC. **(A)** Workflow of single-cell transcriptomic analysis from OSCC patients with different levels of emotional distress before NAIT. **(B)** UMAP plot showing the single cell distribution of patients with different emotional stress. **(C)** UMAP plot showing Leiden clustering of total cells. **(D)** UMAP plot depicting major cell populations across of total cells. **(E)** Dot plot showing canonical marker genes for the identified cell populations. **(F)** UMAP plot of lymphocyte subsets. **(G)** Scatter plot showing the proportions of lymphocyte subsets in ED and No-ED groups. **(H)** Volcano plot showing differentially expressed genes in Treg cells between ED and No-ED groups. **(I, J**). Functional enrichment analysis of Treg cell differential gene signatures across different emotional distress groups. Statistical significance was assessed using two-tailed Student’s t-test or Mann–Whitney U test according to data distribution.

To further investigate the impact of baseline emotional distress on the response to NAIT, we performed single-cell transcriptomic profiling of tumors from OSCC patients with different levels of baseline emotional distress (**Figure 3A**; **Supplementary Table 5**). After quality control and integration, UMAP visualization revealed the overall cellular landscape of the tumor microenvironment and the cell distribution derived from patients with and without emotional distress (**Figure 3B**). Unsupervised Leiden clustering revealed six major cellular populations within the tumor microenvironment, including tumor cells, fibroblasts, endothelial cells, lymphocytes, macrophages, and mast cells (**Figures 3C, D**). Canonical marker genes confirmed the identity of these major cell populations (**Figure 3E**).

Given the central role of lymphocytes in immunotherapy response, we further characterized lymphocyte subsets at single-cell resolution. Reclustering of lymphocytes identified multiple lymphocyte subsets, including regulatory T cells (Treg), cytotoxic, cycling, and naive T cells, as well as plasma cells and multiple B cell populations (**Figure 3F**). Notably, comparison between ED and No-ED groups revealed a significant expansion of Treg cells in tumors from patients with emotional distress, whereas other lymphocyte subsets did not show statistically significant differences (**Figure 3G**; **Supplementary Table 6**). Differential gene expression analysis further demonstrated distinct transcriptional programs in Treg cells between ED and No-ED groups (**Figure 3H**). Functional enrichment analysis revealed that Treg cells from the ED group were significantly enriched in pathways associated with cell proliferation, immune regulation, and cell adhesion. Interestingly, we also observed marked enrichment of myeloid–associated pathways, suggesting potential crosstalk between Treg cells and myeloid populations within the tumor microenvironment (**Figures 3I, J**).

### 3.4 Emotional distress is associated with increased macrophage-Treg crosstalk in the tumor microenvironment

**Figure 4:**
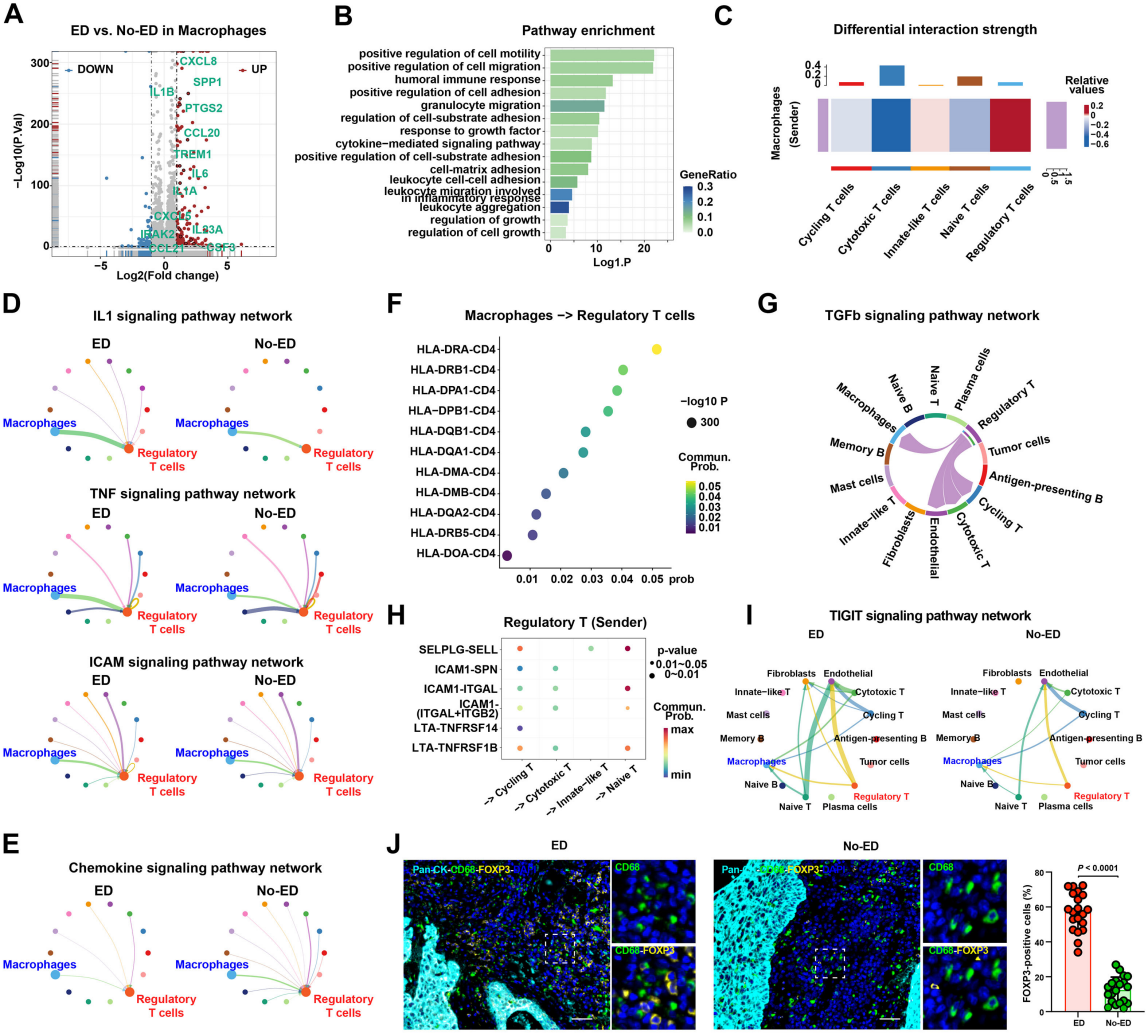
Emotional distress is associated with increased macrophage-Treg crosstalk in the tumor microenvironment. **(A)** Volcano plot showing differentially expressed genes in macrophages between ED and No-ED groups. **(B)** Functional enrichment analysis of macrophage differential gene signatures across different ED groups. **(C)** Cell-cell communication analysis of macrophages with T-cell subsets. **(D, E)** Chord diagrams showing macrophage-Treg interactions in ED and No-ED groups. **(F)** MHC-II signaling interactions from macrophages to Treg cells. **(G)** Chord diagram showing TGF-β signaling originating from Treg cells. **(H)** Dot plot showing regulatory signaling from Treg cells toward lymphocyte populations. **(I)** TIGIT signaling interactions across tumor microenvironmental cells in ED and No-ED groups. **(J)** mIHC showing the spatial distribution of macrophages and Treg cells in the tumor microenvironment of ED and No-ED patients. Statistical significance was assessed using two-tailed Student’s t-test.

To explore the mechanisms underlying Treg expansion in emotionally distressed patients, we next examined macrophage transcriptional changes. Differential gene expression analysis revealed distinct transcriptional profiles in macrophages between ED and No-ED groups (**Figure 4A**). Functional enrichment analysis further showed that ED macrophages were enriched in pathways associated with cell proliferation, immune regulation, and inflammatory responses (**Figure 4B**). Cell-cell communication analysis identified interactions between macrophages and T-cell subsets, particularly between macrophages and Treg cells (**Figure 4C**). Notably, macrophage-Treg interactions mediated by IL1, TNF, and ICAM signaling were markedly increased in the ED group (**Figure 4D**). Interestingly, chemokine–related pathways were more prominent in the No-ED group, suggesting that mechanisms beyond chemotactic recruitment may contribute to Treg enrichment in emotionally distressed tumors (**Figure 4E**). Receptor-ligand analysis further identified strengthened MHC-II signaling interactions from macrophages to Treg cells (**Figure 4F**), while Treg cells exhibited increased TGF-β signaling output toward lymphocyte populations, particularly cytotoxic T cells (**Figure 4G**). In addition, several regulatory ligand-receptor interactions originating from Treg cells were enriched in the ED group, including SELPLG-SELL, ICAM1-SPN, and LTA-TNFRSF1B (**Figure 4H**). Collectively, these signaling interactions were associated with broadly elevated TIGIT-mediated signaling across the tumor microenvironment in the ED group, suggesting a more immunosuppressive immune landscape (**Figure 4I**). Moreover, multiplex immunofluorescence (mIHC) analysis confirmed increased spatial proximity and co-localization of macrophages and Treg cells in tumors from ED patients (**Figure 4J**), supporting increased spatial association between macrophages and Treg cells in emotionally distressed tumors during NAIT.

### 3.5 Stress-associated glucocorticoid signaling is linked to immunosuppressive immune remodeling during NAIT

**Figure 5:**
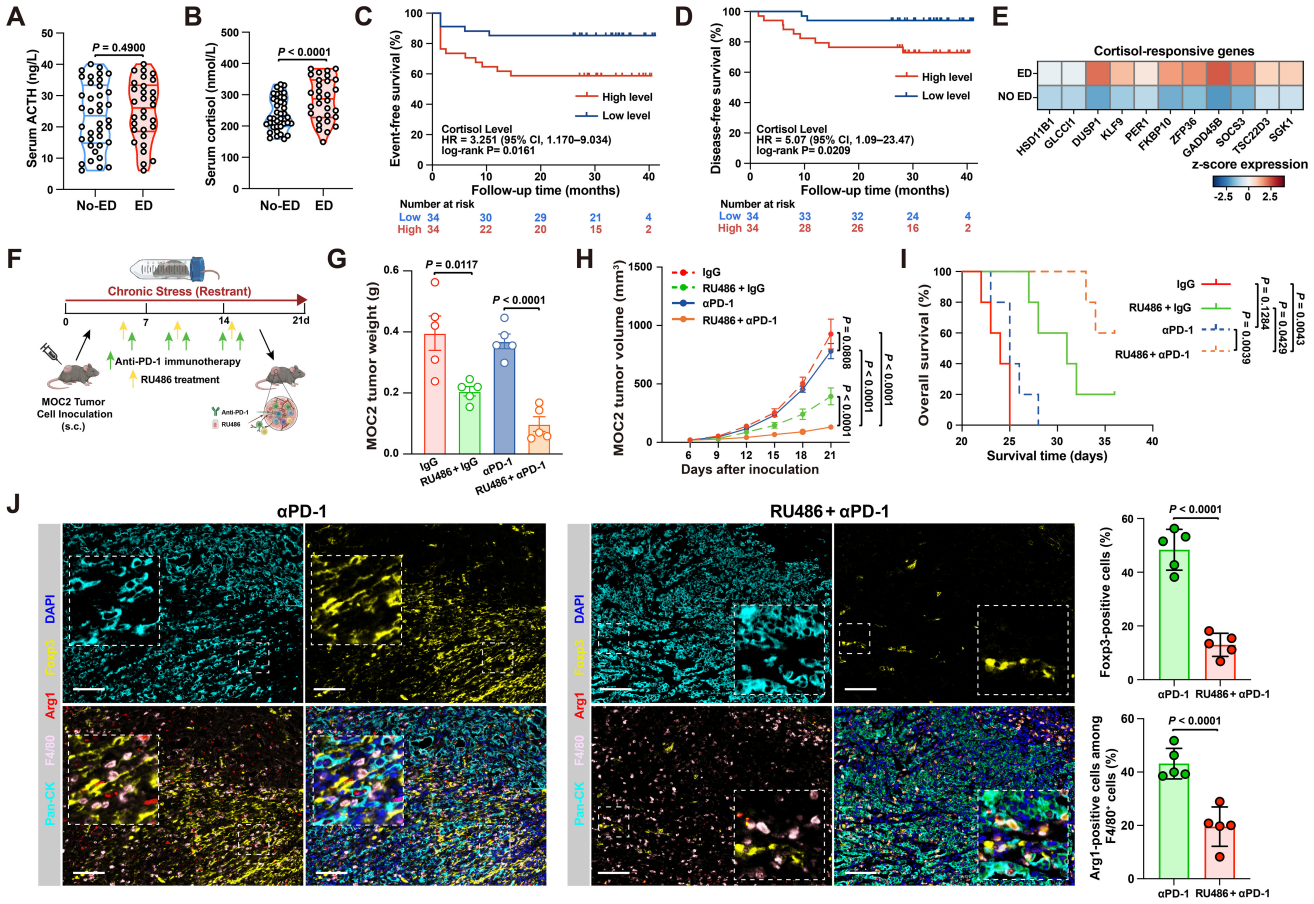
Stress-associated glucocorticoid signaling is linked to immunosuppressive immune remodeling during NAIT. **(A)** Scatter plot showing serum ACTH levels between ED and No-ED groups. **(B)** Scatter plot showing serum cortisol levels between ED and No-ED groups. **(C, D)** Kaplan–Meier analyses of event-free survival and disease-free survival stratified by cortisol level. **(E)** Upregulation of glucocorticoid-related gene expression in patients with emotional distress. **(F)** Schematic overview of the chronic restraint stress mouse model in tumor-bearing mice. **(G, H)** Tumor weight and volume in MOC2 tumor-bearing mice across treatment groups. **(I)** Survival analysis of MOC2 tumor-bearing mice across treatment groups. **(J)** mIHC showing the spatial distribution of immunosuppressive macrophages and Treg cells in tumors from stressed mice treated with or without RU486. Statistical significance for EFS and DFS was assessed using the log-rank test. Group comparisons were performed using two-tailed Student’s t-test or Mann–Whitney U test according to data distribution.

**Figure 6:**
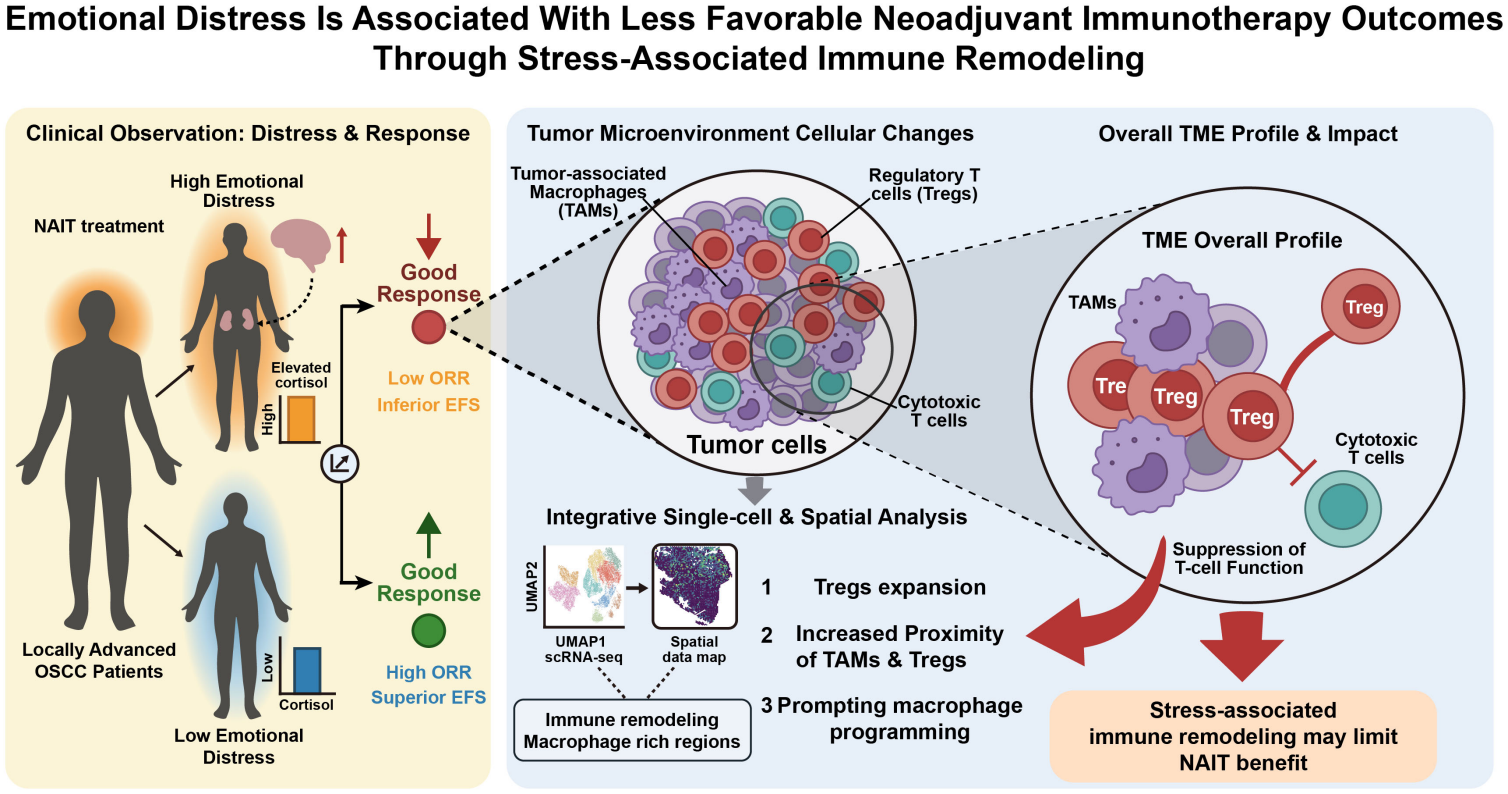
Schematic model of emotional distress-associated immunosuppressive remodeling in OSCC. Emotional distress is associated with neuroendocrine activation and increased glucocorticoid levels, which may contribute to macrophage-associated immunosuppressive remodeling, Treg enrichment, and the formation of immunosuppressive niches linked to less favorable responses to neoadjuvant immunotherapy.

Emotional stress primarily exerts its physiological effects through activation of the sympathetic nervous system and the hypothalamic-pituitary-adrenal (HPA) axis (21, 22). To assess whether stress-associated neuroendocrine activation was linked to NAIT response, we examined key components of these signaling pathways. Serum analysis showed no significant difference in ACTH levels between ED and No-ED patients, whereas cortisol levels were markedly elevated in the ED group (**Figures 5A, B**). Survival analysis further demonstrated that higher cortisol levels were significantly associated with poorer EFS and DFS (**Figures 5C, D**). Consistently, single-cell transcriptomic analysis revealed upregulation of glucocorticoid-responsive genes in tumors from the ED group (**Figure 5E**), suggesting enhanced glucocorticoid-associated signaling within the tumor microenvironment. Notably, treatment with RU486, a glucocorticoid receptor antagonist (23), appeared to reduce tumor burden and improve survival during anti-PD-1 therapy in the mouse stress model (**Figures 5F–I**). In addition, mIHC analysis showed a trend toward reduced accumulation of immunosuppressive macrophages and Tregs in tumors after RU486 treatment (**Figure 5J**). The mIHC further suggested that RU486 treatment was associated with decreased proliferative activity of Treg cells located near macrophages in stressed mice (**Supplementary Figure 3A**). To further assess macrophage–Treg crosstalk-related molecular programs, macrophages and Treg cells were isolated from mouse tumor tissues by flow cytometry, followed by qPCR analysis of IL1-, TNF-, ICAM-, and CXCL-related genes (**Supplementary Figure 3B**). RU486 treatment was associated with changes in macrophage-expressed ligand genes, suggesting partial modulation of stress-associated inflammatory and adhesion programs. Collectively, these findings suggest that emotional distress may be linked to less favorable NAIT outcomes through stress-associated remodeling of the tumor immune microenvironment, with glucocorticoid signaling representing a potential contributing mechanism (**Figure 6**).

## 4 Discussion

Increasing evidence suggests that OSCC involves systemic interactions across multiple organs and biological levels (24). Multi-layered investigations integrating tumor genomics, immune profiling and systemic physiology have already led to meaningful clinical advances. Comprehensive immune characterization of the tumor microenvironment has facilitated the NAIT development, while multi-omics analyses have enabled the identification of predictive biomarkers for patient stratification (25, 26). However, the role of host psychological factors as systemic modulators of antitumor immunity remains largely unexplored. In this study, we systematically investigated the association between emotional distress and NAIT outcomes in patients with locally advanced OSCC. By integrating psychological assessment, serological profiling, and tumor microenvironment analyses, we identified a significant association between emotional distress and clinical prognosis. Patients with elevated emotional distress showed a lower disease control rate compared with those without distress, a difference that extended beyond short-term therapeutic outcomes and was reflected in event-free survival. These clinical findings prompted further exploration into the underlying biological features linking psychological stress to immunotherapeutic resistance.

Emotional distress has emerged as a host-related factor associated with tumor progression and therapeutic responsiveness (27). Increasing evidence suggests that its immunological impact is mediated, at least in part, through activation of the hypothalamic-pituitary-adrenal (HPA) axis and downstream glucocorticoid signaling (20, 28). In line with this framework, patients with emotional distress in our cohort exhibited significantly elevated serum cortisol levels, reflecting heightened neuroendocrine activation (29). This endocrine alteration was associated with less favorable immunotherapeutic outcomes and accompanied by distinct immunological features. Concurrently, the tumor microenvironment in the ED group displayed a dysregulated phenotype characterized by increased FOXP3+ regulatory Tcell infiltration, which may diminish effector immune cell infiltration as previously reported. Moreover, serum cortisol levels were inversely correlated with therapeutic response, further supporting a potential association between neuroendocrine activation and immunotherapy resistance. In OSCC animal model, RU486 treatment was associated with favorable changes in tumor burden and survival in the mouse stress model, providing supportive pharmacological evidence for the potential involvement of glucocorticoid-associated signaling. These observations point to a stress-associated neuroimmune axis that may affect treatment efficacy in OSCC.

Neuroendocrine perturbations associated with emotional distress are increasingly recognized as potential contributors to tumor microenvironment remodeling (30, 31). Among immune populations, myeloid cells appear to be particularly sensitive to stress-associated neuroendocrine signaling (11, 32). Previous studies have shown that glucocorticoids can profoundly influence macrophage differentiation, polarization, and cytokine production, thereby reshaping immune communication networks within tumors and promoting immunoregulatory phenotypes (33–35). Consistent with this concept, our single-cell and cell-cell communication analyses revealed enhanced macrophage-Treg interactions in tumors from emotionally distressed patients. Notably, macrophage-derived signaling pathways associated with immune activation and adhesion were enriched, whereas chemokine-related pathways were not increased, suggesting that Treg enrichment under distress conditions may involve macrophage–associated proliferation or retention, although chemotactic recruitment and other mechanisms cannot be excluded. Spatial multiplex immunofluorescence further demonstrated pronounced co-localization of macrophages and Tregs at the tumor-stroma interface, forming localized immunosuppressive niches characterized by increased macrophage abundance and Treg infiltration. Similar spatially organized suppressive domains have been described in multiple solid tumors, where macrophage-Treg cooperation contributes to immune exclusion and impaired antitumor immunity (36, 37). Such spatial organization may restrict cytotoxic lymphocyte access to tumor cells and reduce local interferon signaling, thereby limiting PD-L1 expression and reinforcing a “pseudo-cold tumor” phenotype (38). Together, these findings suggest that emotional distress may be associated with tumor immune remodeling involving macrophage-Treg spatial organization, which could contribute to localized immunosuppression and reduced antitumor immunity.

However, this study has several limitations. First, although we performed multivariable and sensitivity analyses, residual confounding could not be fully excluded. In particular, incomplete capture of cortisol-related confounders limited our ability to perform a reliable fully adjusted analysis of cortisol and treatment outcomes. Second, the EORTC QLQ-C30 emotional functioning subscale has established clinical thresholds and has been used in prior NAIT studies, but it is not a structured psychiatric assessment. Thus, ED in this study should be interpreted as impaired patient-reported emotional functioning rather than a formal psychiatric diagnosis. Although our eligibility criteria excluded several major confounders, including systemic corticosteroid or psychotropic medication use, disease-related symptoms and tumor burden cannot be fully separated from emotional functioning in locally advanced OSCC. This limitation also highlights the clinical importance of recognizing and assessing emotional impairment in OSCC patients. Third, although single-cell, spatial, multiplex immunofluorescence, and animal data support stress-associated immune remodeling and macrophage–Treg crosstalk, further functional studies are needed to establish causality. Finally, the female subcutaneous MOC2 stress model and RU486 intervention do not fully recapitulate human oral mucosal OSCC or GR-specific signaling. Future studies using larger independent cohorts, orthotopic OSCC models, more specific GR-targeting approaches, and prospective psychosocial intervention designs are needed.

In conclusion, our study suggests that emotional distress is associated with less favorable NAIT outcomes in OSCC and with immune remodeling within the tumor microenvironment. Emotional distress was linked to regulatory T cell enrichment and increased macrophage-Treg spatial interactions, consistent with the formation of immunosuppressive tumor niches. These findings extend current immunotherapy stratification paradigms beyond tumor-intrinsic factors and highlight the potential value of incorporating emotional assessment and psychosocial intervention into neoadjuvant treatment strategies for OSCC.

## Data Availability

The datasets presented in this study can be found in online repositories. The names of the repository/repositories and accession number(s) can be found in the article/**Supplementary Material**.
