# Supplementary material for: Emotional distress is associated with neuroendocrine-immune remodeling and less favorable neoadjuvant immunotherapy outcomes in oral squamous cell carcinoma
Source: Front Immunol. 2026 Jul 1;17:1861003. doi: 10.3389/fimmu.2026.1861003 (PMC13369479; doi:10.3389/fimmu.2026.1861003)
Supplement: Supplementary file 1 [file DataSheet1.docx]

Supplementary Material

# Supplementary Figures and Tables

## Supplementary Figures

##
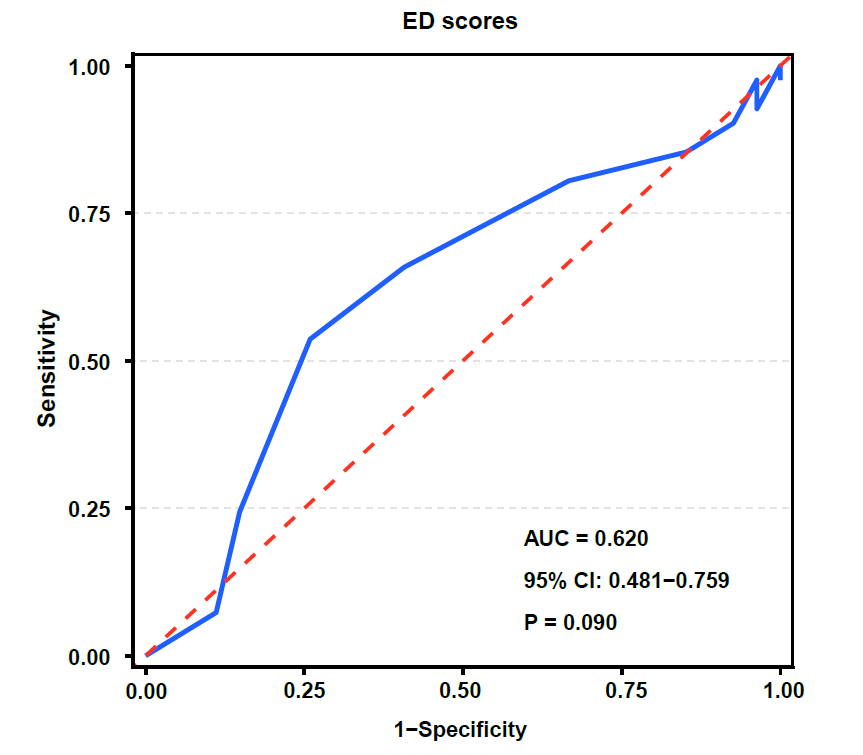


**Supplementary Figure 1. ROC analysis of the baseline emotional functioning score for discriminating treatment response.**

**
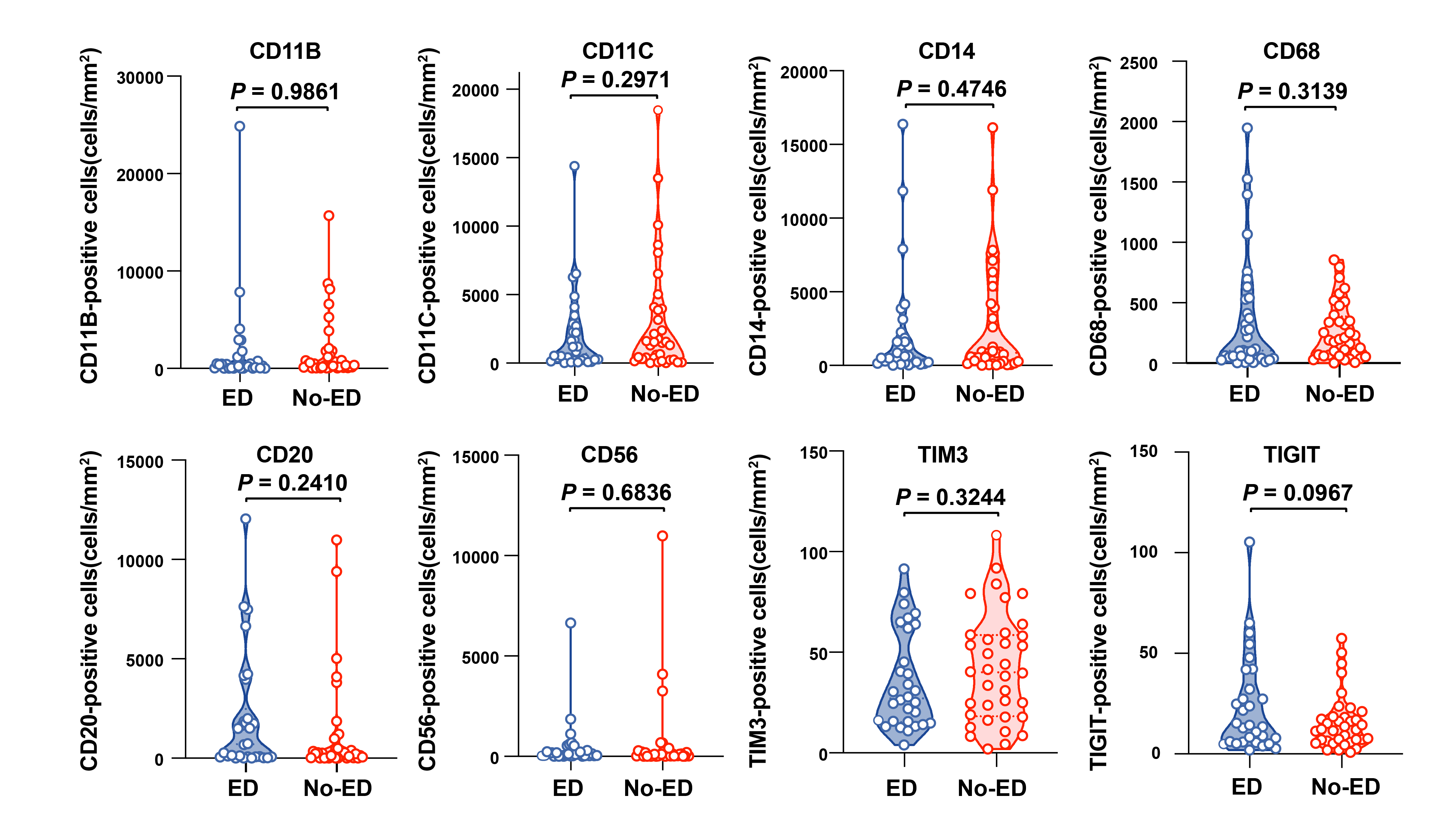
**

**Supplementary Figure 2. Quantitative comparison of immune cell infiltration between ED and No-ED groups.**


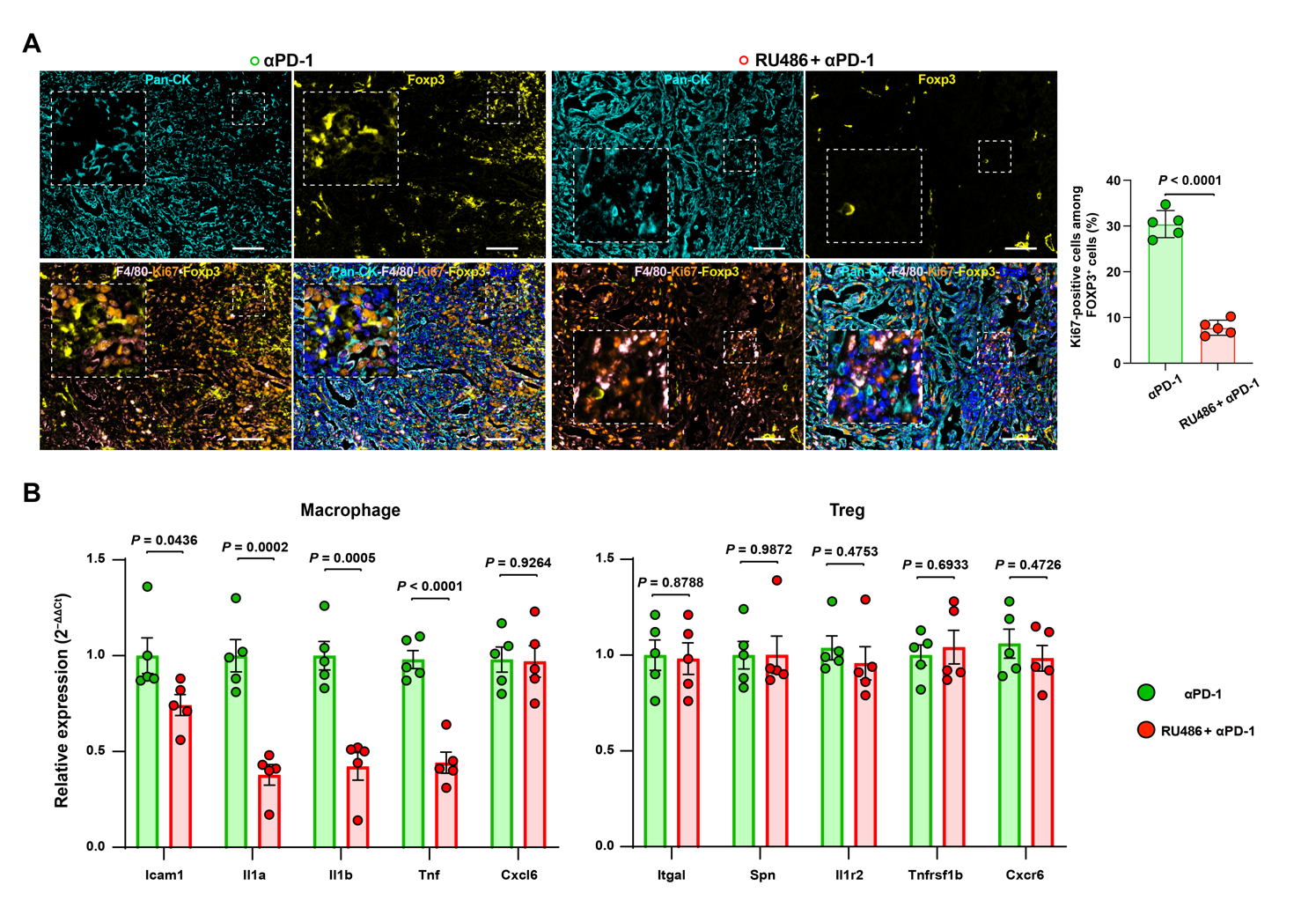


**Supplementary Figure 3. RU486 treatment partially attenuates stress-associated macrophage-Treg interactions and Treg proliferative activity. A.** RU486 treatment was associated with reduced proliferative activity of Treg cells located near macrophages in stressed mice. **B**. qPCR analysis of the interaction-related genes after RU486 treatment in the stressed mouse model.

| **Baseline Characteristics of the 68 Enrolled Patients receiving NAIT** | | | | | |
| --- | --- | --- | --- | --- | --- |
|  | **No. (%)** | | | |  |
| **Characteristic** | **Overall**  **(n = 68)** | **ED**  **(n = 30)** | | **No-ED**  **(n = 38)** | ***P* value** |
| **Age at enrollment, years** |  |  | |  |  |
| Mean (range) | 50.7 (32.0 – 68.0) | 49.7 (32.0 – 67.0) | | 51.7 (33.0 – 68.0) |  |
| **Gender** |  | |  |  | 0.493 |
| Male | 59 (86.8) | 25 (83.3) | | 34 (89.5) |  |
| Female | 9 (13.2) | 5 (16.7) | | 4 (10.5) |  |
| **Smoking history** |  |  | |  | 0.794 |
| Current or former | 47 (69.1) | 20 (66.7) | | 27 (71.1) |  |
| Never | 21 (30.9) | 10 (33.3) | | 11 (28.9) |  |
| **Alcohol use history** |  |  | |  | 1 |
| Current or former | 32 (47.0) | 14 (46.7) | | 18 (47.4) |  |
| Never | 36 (53.0) | 16 (53.3) | | 20 (52.6) |  |
| **Betel nut use history** |  |  | |  | 0.112 |
| Current or former | 20 (29.4) | 12 (40.0) | | 8 (21.1) |  |
| Never | 48 (70.6) | 18 (60.0) | | 30 (78.9) |  |
| **ECOG performance status** |  |  | |  | 1 |
| 0 | 44 (64.7) | 19 (63.3) | | 25 (65.8) |  |
| 1 | 24 (35.3) | 11 (36.7) | | 13 (34.2) |  |
| **Tumor site** |  |  | |  | 0.306 |
| Oral tongue | 35 (51.5) | 14 (46.7) | | 21 (55.3) |  |
| Gingiva | 8 (11.8) | 6 (20.0) | | 2 (5.3) |  |
| Floor of mouth | 9 (13.2) | 4 (13.3) | | 5 (13.2) |  |
| Buccal mucosa | 16 (23.5) | 6 (20.0) | | 10 (26.3) |  |
| **Clinical T-stage^a^** |  |  | |  | 0.514 |
| T2 | 15 (22.1) | 8 (26.7) | | 7 (18.4) |  |
| T3 | 37 (54.4) | 14 (46.7) | | 23 (60.5) |  |
| T4 | 16 (23.5) | 8 (26.7) | | 8 (21.1) |  |
| **Clinical N-stage^a^** |  |  | |  | 0.808 |
| N0 | 36 (53.0) | 17 (56.7) | | 19 (50.0) |  |
| N1 | 16 (23.5) | 6 (20.0) | | 10 (26.3) |  |
| N2 | 16 (23.5) | 7 (23.3) | | 9 (23.7) |  |
| **Clinical disease stage** |  |  | |  | 1 |
| III | 39 (57.4) | 17 (56.7) | | 22 (57.9) |  |
| IVA | 29 (42.6) | 13 (43.3) | | 16 (42.1) |  |
| **Treatment** |  |  | |  | 0.087 |
| Monotherapy | 34 (50.0) | 11 (36.7) | | 23 (60.5) |  |
| Combination | 34 (50.0) | 19 (63.3) | | 15 (39.5) |  |
| **Hypertension** |  |  | |  | 1 |
| No | 49 (72.1) | 22 (73.3) | | 27 (71.1) |  |
| Yes | 19 (27.9) | 8 (26.7) | | 11 (28.9) |  |
| **Diabetes** |  |  | |  | 0.721 |
| No | 59 (86.8) | 27 (90.0) | | 32 (84.2) |  |
| Yes | 9 (13.2) | 3 (10.0) | | 6 (15.8) |  |
|  | | | | |  |

**Supplementary Table 1. Baseline characteristics of the 68 enrolled patients receiving NAIT.**

Abbreviation: ECOG, Eastern Cooperative Oncology Group,

a American Joint Committee on Cancer, 8th Edition staging.


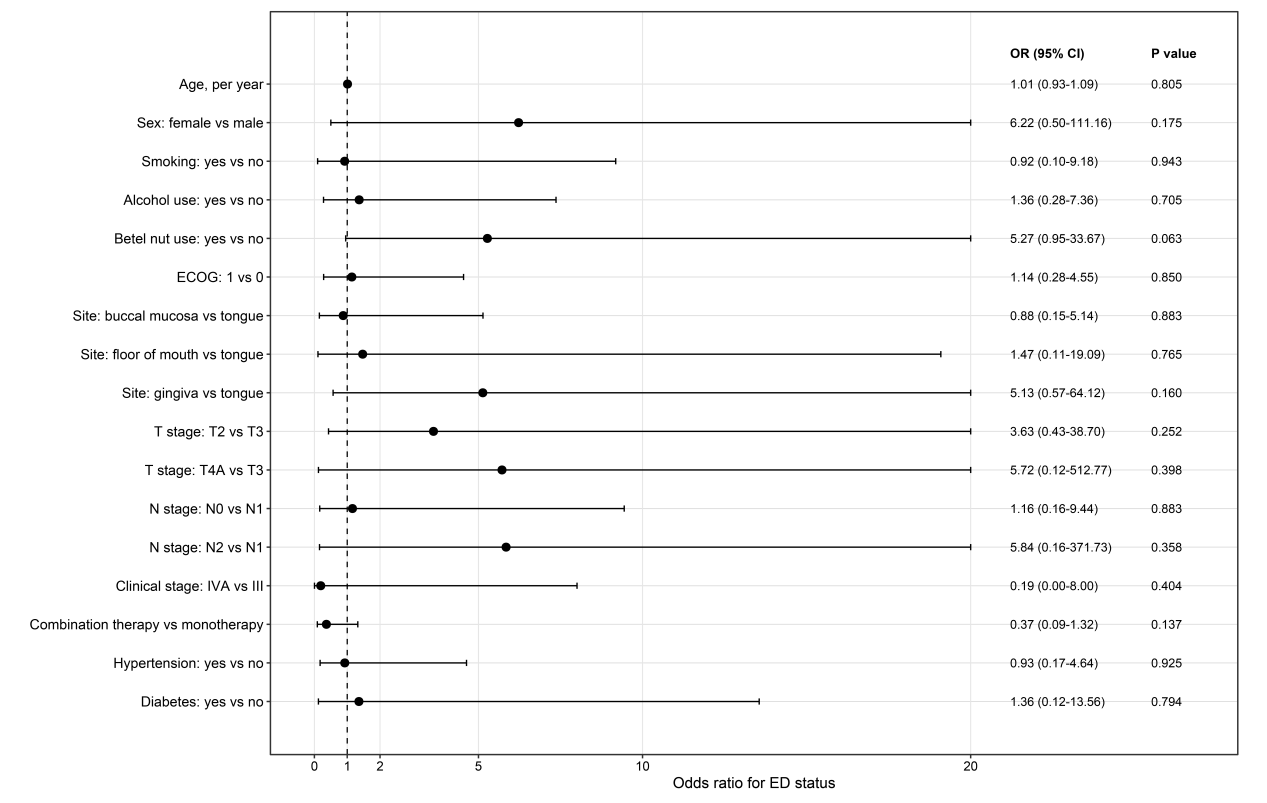


**Supplementary Table 2. Multivariable logistic regression analysis of factors associated with emotional distress.**

**
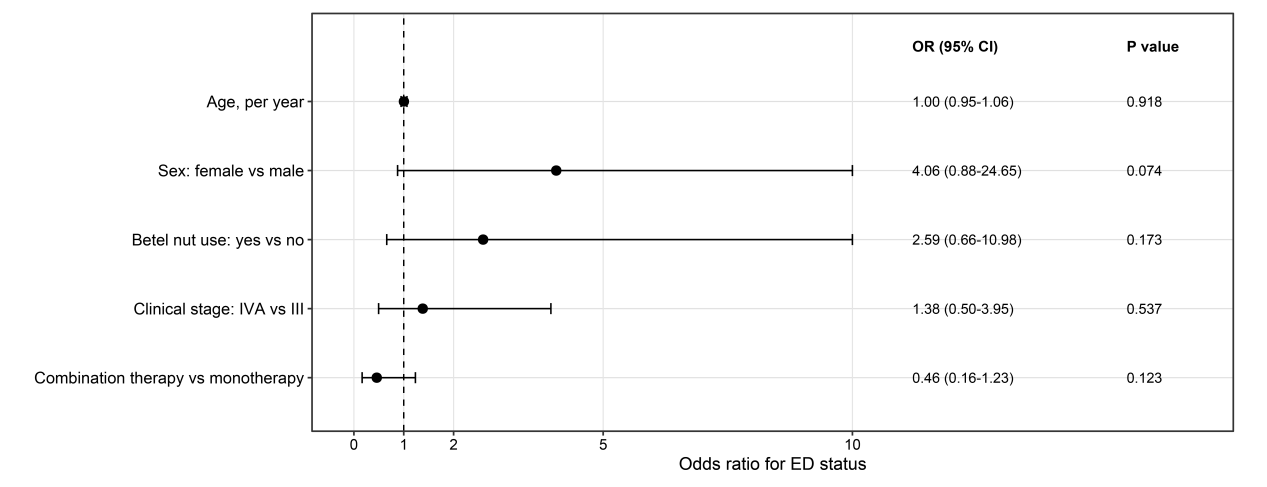
**

**Supplementary Table 3. Penalized logistic regression analysis of factors associated with emotional distress.**


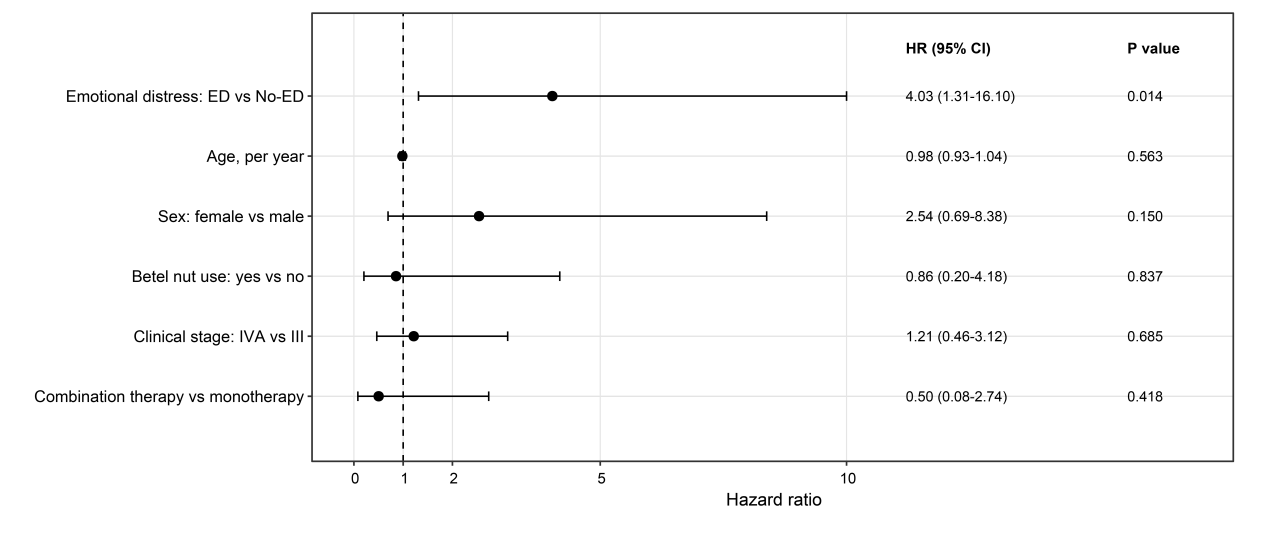


**Supplementary Table 4. Multivariable Cox regression analysis of emotional distress and event-free survival.**

| **Id** | **Group** | **Batch** | **Time** | **Cells** | **Median genes per cell** | **Median UMIs per cell** | **Median mito percent,%** |
| --- | --- | --- | --- | --- | --- | --- | --- |
| **P1** | **No-ED** | **B1** | **Pre-NAIT** | **6220** | **2560** | **9043** | **12.152** |
| **P2** | **ED** | **B2** | **Pre-NAIT** | **5805** | **1659** | **4933** | **8.881** |
| **P3** | **ED** | **B3** | **Pre-NAIT** | **5583** | **2026** | **6395** | **7.523** |
| **P4** | **No-ED** | **B4** | **Pre-NAIT** | **9486** | **2871.5** | **10230** | **13.584** |
| **P5** | **ED** | **B5** | **Pre-NAIT** | **5783** | **2044** | **6565.5** | **8.594** |
| **P6** | **ED** | **B6** | **Pre-NAIT** | **7355** | **2138** | **6889** | **12.182** |
| **P7** | **No-ED** | **B7** | **Pre-NAIT** | **7267** | **2372.5** | **6491** | **7.017** |
| **P8** | **ED** | **B8** | **Pre-NAIT** | **10132** | **2595.5** | **8557.5** | **9.381** |
| **P9** | **No-ED** | **B9** | **Pre-NAIT** | **9086** | **1010** | **2803** | **13.99** |
| **P10** | **ED** | **B10** | **Pre-NAIT** | **8329** | **1758** | **4551** | **6.413** |
| **P11** | **No-ED** | **B11** | **Pre-NAIT** | **13238** | **2680** | **8335.5** | **15.723** |
| **P12** | **ED** | **B12** | **Pre-NAIT** | **9034** | **1771** | **4785.5** | **8.308** |
| **P13** | **ED** | **B13** | **Pre-NAIT** | **8018** | **1739** | **6165** | **6.613** |
| **P14** | **No-ED** | **B14** | **Pre-NAIT** | **3908** | **2136** | **6751** | **12.876** |
| **P15** | **No-ED** | **B15** | **Pre-NAIT** | **6962** | **1156** | **2765.5** | **8.204** |
| **P16** | **No-ED** | **B16** | **Pre-NAIT** | **4682** | **1233** | **3172** | **13.325** |
| **P17** | **No-ED** | **B17** | **Pre-NAIT** | **4705** | **1755** | **4932** | **8.354** |

**Supplementary Table 5. Patient-level metadata and scRNA-seq quality control metrics.**

| **Feature** | **Mean proportion (ED, %)** | **Mean proportion (No-ED, %)** | **P value** |
| --- | --- | --- | --- |
| **Cycling T cells** | **1.858** | **2.469** | **0.674** |
| **Cytotoxic T cells** | **2.794** | **0.237** | **0.134** |
| **Innate-like T cells** | **0.044** | **0.781** | **0.384** |
| **Memory B cells** | **1.934** | **0.568** | **0.302** |
| **Naive B cells** | **2.581** | **0.147** | **0.232** |
| **Naive T cells** | **0.025** | **3.701** | **0.309** |
| **Plasma cells** | **4.042** | **1.164** | **0.260** |
| **Regulatory T cells** | **5.887** | **0.175** | **0.028** |
| **Antigen-presenting B cells** | **0** | **1.261** | **0.340** |

**Supplementary Table 6. Differential abundance of immune cell subsets.**

| **Endpoint** | **Group** | **n** | **Events/censored, n** | **HR (95% CI)** | **P value** |
| --- | --- | --- | --- | --- | --- |
| **EFS** | **No-ED** | **38** | **6/32** | **Reference** | **-** |
| **EFS** | **ED** | **30** | **13/17** | **3.083 (1.237-7.685)** | **0.0116** |

**The median follow-up time was 30.24 months**

**Supplementary Table 7. Event-free survival analysis in the overall patient cohort.**
